# Tissue-Plasma TMB Comparison and Plasma TMB Monitoring in Patients With Metastatic Non-small Cell Lung Cancer Receiving Immune Checkpoint Inhibitors

**DOI:** 10.3389/fonc.2020.00142

**Published:** 2020-02-12

**Authors:** Alex Friedlaender, Thierry Nouspikel, Yann Christinat, Liza Ho, Thomas McKee, Alfredo Addeo

**Affiliations:** ^1^Department of Oncology, University Hospital of Geneva (HUG), Geneva, Switzerland; ^2^Service of Medical Genetics, Diagnostics Department, University Hospital of Geneva, Geneva, Switzerland; ^3^Department of Pathology, University Hospital of Geneva, Geneva, Switzerland

**Keywords:** CtDNA, immune check inhibitor (ICI), biomarker, TMB, NSCLC

## Abstract

Immuno-oncology is an ever growing field that has seen important progress across the spectrum of cancers. Responses can be deep and durable. However, as only a minority of patients respond to checkpoint inhibition, predictive biomarkers are needed. Cancer is a genetic disease arising from the accumulation of somatic mutations in the DNA of affected cells. Tumor mutational burden (TMB), represents the number of somatic mutations in a tumor that form neoantigens, responsible for the immunogenicity of tumors. Randomized controlled trials have so far failed to show a survival benefit when stratifying patients by tissue TMB. TMB has also been evaluated in plasma (PTMB). PTMB is anticipated to represent the biology of the entire cancer, whereas obtaining tissue of an amenable primary or a metastatic lesion may be prone to sampling bias because of tumor heterogeneity. For this reason, we are evaluating the correlation between TMB and PTMB, and prospectively evaluating the impact of these biomarkers on clinical outcomes. We also discuss the technical difficulties inherent to performing and comparing these analyses. Furthermore, we evaluate the correlation between the evolution of PTMB during an immunotherapy treatment and response at 3 and 6 months, as we believe PTMB may be a dynamic biomarker. In this paper, we present results from the first 4 patients in this project.

## Introduction

Immuno-oncology is an ever growing field that has seen important progress across the spectrum of cancers. While results have changed the prognosis for certain types of tumors, including melanoma, lung, genito-urinary, and digestive cancers, only a minority of patients responds to treatment and reaps these benefits. Different biomarkers have been developed with the goal of predicting response to treatment (1). Yet, only one has reached the maturity needed for clinical utility, programmed death ligand-1 (PD-L1). PD-L1 expression, assessed on tumor cells and immune-cells, correlates with response rate and survival in non-small-cell lung cancer (NSCLC).

In front line management of advanced NSCLC, the Keynote 024 trial concluded that patients with tumors expressing ≥50% PD-L1 derive greater clinical outcomes with pembrolizumab, a PD-1 immune checkpoint inhibitor (ICPI), than with platinum-based chemotherapy, both in terms of response rate (44. vs. 27.8%) and overall survival (OS), with a 3 year OS of 43% (2). The Keynote 042 trial went on to show front-line ICPI activity in all PD-L1 subsets of NSCLC, though higher PD-L1 expression is predictive of response (3).

The 5 year overall survival data of the Keynote 001 trial, presented at the American Society for Clinical Oncology (ASCO) 2019 conference, revealed durable long-term responses to immune-checkpoint inhibition (ICPI), and an unprecedented 5-year OS of 13.4–29.6% (4–6). This is a drastic improvement over pre-ICPI 5 year survival rates, which were below 5% (7).

However, these results also underline that despite being exposed to potential adverse effects of therapy, the majority of patients do not derive a durable benefit from ICPI, even in the selected high PD-L1 population. This highlights the need for further predictive biomarkers for ICPI.

It is known that cancer is a genetic disease and that neoplastic transformation results from the accumulation of somatic mutations in the DNA of affected cells. The genetic modifications in tumors can include non-synonymous mutations comprising mainly missense mutations, as well as synonymous mutations, insertions or deletions, splice site mutations and copy number gains and losses. Tumor mutational burden (TMB), represents the number of somatic mutations in a tumor, but there is no consensus as to which mutations should be included in the calculation: some authors report all mutations (8, 9), others use only non-synonymous mutations (10), and yet others (11–13) consider only mutations that alter the sequence of a protein (i.e., missense and indels within exons). The former vary in prevalence between 0.01 mutations/megabase pair (Mbp) and 400 mutations/Mbp. In this paper, we elected to report miscoding mutations, i.e., mutations that yield the translation of novel peptide epitopes, since it is thought that neoantigens are responsible for the immunogenicity of the tumor by eliciting T cell responses. This is the basis for the hypothesis that higher TMB should allow for greater benefit from ICPI.

This hypothesis has some clinical data in its favor. For instance, tumors known, through DNA sequencing, to harbor multiple acquired mutations, such as microsatellite unstable tumors, melanoma and non-small-cell lung cancer, are those with the best response to ICPI (8). Furthermore, studies have shown improved response rates (RRs) and progression free survival (PFS) in patients with tumors deemed to have high tissue TMB (9, 11). Yet, there appears to be no correlation between OS with single-agent ICPI and TMB in NSCLC, while the predictive value of TMB in combined PD-1 blockade and anti- cytotoxic TIL antigen-4 (CTLA4) inhibition showed promising PFS data (14, 15). Unfortunately, the recently published Checkmate 227 trial has now negated these early results, showing no association between TMB and OS (12).

TMB has also been evaluated in plasma (PTMB). PTMB is anticipated to represent the biology of the entire cancer, whereas obtaining tissue of an amenable primary or a metastatic lesion may be prone to sampling bias because of tumor heterogeneity (16). However, while tissue samples can be microdissected to yield a high percentage of tumor cells, circulating cell free DNA (ccfDNA) can be more challenging to interpret in this regard. Identifying circulating tumor DNA (ctDNA), which can be present as a minute fraction of total ccfDNA, can be limiting for next-generation sequencing (NGS) analysis (17). In a recent publication comparing TMB and PTMB by whole exome sequencing in various cancer types, the sensitivity for mutation detection was 53% in ccfDNA. The lower sensitivity was, as expected, often associated with lower ctDNA levels. Meanwhile, the sensitivity of ctDNA NGS compared to WES was 92%, suggesting that NGS may be a valid surrogate detection method, like in tissue (13).

Important clinical questions remain to be answered: is there a correlation between NGS-based TMB and PTMB in NSCLC? Will one be a stronger predictor of response to immunotherapy?

For this reason, we are evaluating the correlation between TMB and PTMB, and prospectively evaluating the impact of these biomarkers on clinical outcomes. We also discuss the technical difficulties inherent to performing and comparing these analyses. Furthermore, we evaluate the correlation between the evolution of PTMB during an immunotherapy treatment and response at 3 and 6 months, as we believe PTMB may be a dynamic biomarker. In this paper, we present results from the first 4 patients in this project.

## Methods

### Patients

We selected 4 consecutive stage IV NSCLC patients before the introduction of pembrolizumab, an anti-PD-1 checkpoint inhibitor. Patients and tumor characteristics were collected from medical records, pathology, and radiology reports.

### Samples

Blood was drawn and collected in Streck tubes before treatment and after 3 months of treatment with pembrolizumab. Plasma was prepared by centrifugation 10 min at 1,600 × g, collected, spun again at 16,000 × g for 10 min, and stored at −80°C until used. The tissue sample was collected at diagnosis.

### DNA Extraction

Cell-free DNA was prepared from 4 to 5 ml plasma with the MinElute ccfDNA kit (Qiagen) or the Cobas cfDNA kit (Roche) according to the manufacturer instructions. Tumor DNA was prepared from formalin-fixed paraffin-embedded (FFPE) samples using the QIAamp DNA FFPE Tissue kit (Qiagen). Germline DNA was extracted from whole blood with QIAamp DNA blood mini kit (Qiagen). DNA was stored at −20°C until used.

### Sequencing

A custom 443-gene, 2 073 Mbases SureSelect-HS library (Agilent) was built from 10 ng ccfDNA or 50 ng genomic DNA. Paired-end sequencing, 2 × 150 nt, was performed on a NextSeq500 sequencer (Illumina).

### Analysis

Data from plasma samples were preprocessed with the AGeNT package (Agilent) for molecular tag deduplication, then variants were called with SiNVICT (18) retaining level 5 variants, LoFreq (19) v 2.1.2, single sample mode with parameter *a* = 10–9 for plasma samples, OutLyzer (20) v 2.0, “calling” command, with default parameters or SNVer (21) v 0.5.3 with default parameters. For PTMB calculations, only miscoding variants (exonic variants with the potential of modifying the protein sequence: missense, non-sense, and indels), with a frequency <30% and absent from whole blood DNA were counted, the total was normalized to the total size of the regions sequenced. Tumor data was analyzed with a combination of Strelka (22) v 2.9.6 and MuTect2 (23) v 4.1.0.0, only variants called by both callers, with frequency >2%, frequency in tumor >4-fold higher than in normal tissue and average base quality >20 were retained. For TMB calculations, only miscoding variants were considered, for tumor data in table I only mutations with a frequency >5% were counted. For **Figure 3**, tumor data was re-analyzed with SiNVICT, using the same parameters and same counting criteria as for PTMB, for the sake of comparison.

### Outcomes

Response was evaluated radiologically using the immune RECIST criteria (24) and clinically. Progression was defined as radiologic progression or the appearance of new cancer related symptoms or death. Time to treatment failure was calculated from the start of immunotherapy to its interruption due to progression requiring subsequent systemic therapy.

## Results

### Patients

Of the four included in this preliminary analysis, three had adenocarcinoma, one squamous cell carcinoma. Three patients were male, one female. No patients harbored any druggable driver mutations, analyzed by next-generation sequencing. Three patients had a high PD-L1 expression (above 50%). The age range spanned from 66 to 74 years old (Table 1).

**Table 1:** Patient and tumor characteristics.

**Patient**	**Age**	**Sex**	**Histology**	**PD-L1 (%)**	**TMB (mut/mbp)**	**Time 0 PTMB (mut/Mbp)**	**3 month PTMB (mut/Mbp)**	**ICPI**	**Line**	**3 month response**	**6 month response**	**TTF**
PIT-063	74	Male	ADC	90	6.3	1.4	0.0	Pembro	1	Response	Response	14 (ongoing)
PIT-069	66	Male	ADC	75	8.7	2.9	0.5	Pembro	1	Response	Dissociated	15
PIT-075	66	Male	Squamous	10	3.4	5.8	9.6	Pembro	2	Response	/	14
PIT-077	67	Female	ADC	70	1.4	4.3	12.1	Pembro	1	Progression	Death	4

*ADC, adenocarcinoma; PD-L1, programmed death ligand-1; TMB, tumor mutation burden; PTMB, plasma mutation burden; Pembro, pembrolizumab; ICPI, immune checkpoint-inhibitor; TTF, time to treatment failure; Mut/Mbp, mutations per megabase pair*.

### Amount of Cell-Free DNA

**Figure 1:**
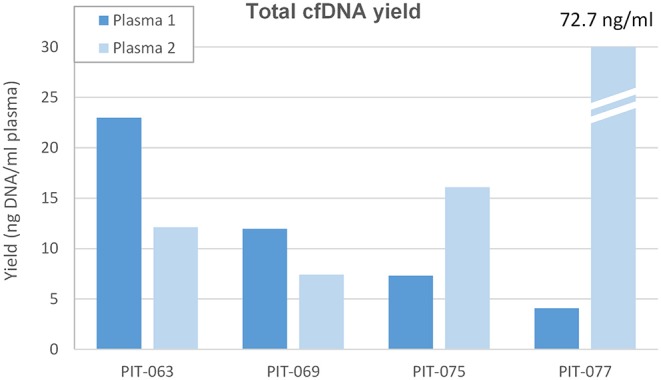
Total cell-free DNA yield. Amount of DNA removed from the first (Plasma 1, concomitant to surgery) and second (Plasma 2, after 3 months therapy) plasma samples, expressed as ng ccfDNA per ml plasma.

We measured the amount of cell-free DNA recovered and normalized it to the amount of plasma processed (Figure 1). In most cases, cell-free DNA yield matched the values we routinely obtain with healthy controls, around 10 ng/ml plasma. Patient PIT-063 had significantly higher cell-free DNA levels at the time of the first blood draw (23 ng/ml plasma), but this remains within the physiological range, as it is well-known that cell-free DNA levels can vary considerably for a given individual, depending, for instance on physical exercise or even psychosocial stress (25). Patient PIT-77 displayed low amounts of cell-free DNA at the first blood draw, but very high levels at the second, with an almost 100-fold increase over a 3-month period. We assumed that, in this case, the increase was due to a higher amount of ctDNA, which was confirmed by mutation analysis (see below).

### Correlation Between Mutations Found in Tumor and Plasma

Bioinformatic identification of somatic mutations in sequencing data is a two-step process. During the initial, straightforward step, individual sequence reads are aligned to the human reference genome, and divergences from the reference (variants) are collated. If molecular barcodes are used, reads can be aggregated based on the original DNA molecule they were amplified from, allowing for some level of error polishing (26). The second and most critical step is to classify variants: some will be germline polymorphisms that are easy to recognize as variant frequencies will approach 50 or 100%, depending on whether the patient is heterozygous or homozygous. The main difficulty lies in the interpretation of low frequency variants, some of which correspond to genuine somatic mutations, while others are background noise, i.e., PCR errors or sequencing mistakes. This filtering process is particularly crucial in the context of mutation burden analysis which essentially tallies passenger mutations, most of which will have appeared in subclones of the tumor. As a result, mutation frequencies are expected to be low in the tumor and even lower in plasma, owing to dilution of ctDNA with ccfDNA from normal cells.

Numerous software packages have been proposed to sift somatic mutations from background noise (27). We extensively tested several, based on different underlying algorithms: SiNVICT (18) (Poisson model with additional heuristic filters), OutLyzer (20) (noise level estimation by recursive Thompson tau tests), SNVer (21) (allele frequency analysis by binomial-binomial model) and LoFreq (19) (allele frequency analysis by Poisson-binomial model). To evaluate the performance of these programs, we used the mutations identified in the tumor as a “truth set” and asked how many of these could be detected in plasma and identified as somatic mutation by a given variant caller.

Most mutations found in the tumor were also present in the corresponding plasma samples, albeit at low frequencies (<3%). As a result, none of the software packages we tested succeeded in calling a major fraction of these mutations. In our hands, the best programs were SiNVICT and LoFreq, the latter slightly more performant. For this reason, the data presented hereafter were produced with LoFreq, although analysis with SiNVICT led to identical conclusions.

**Figure 2:**
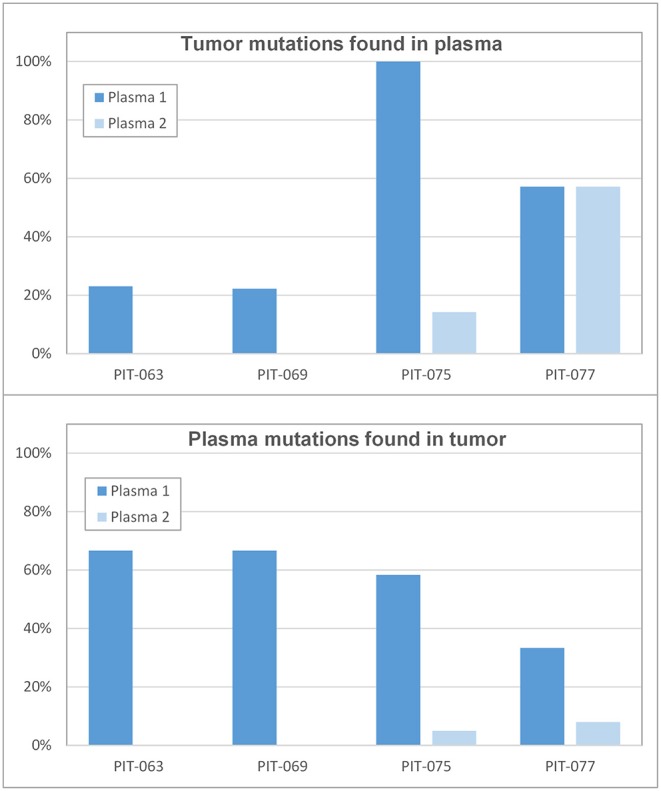
Correlation between tumor and plasma samples (**Top**) percent of tumor mutations that were found in the first (time 0) and second (3 months) plasma samples. (**Bottom**) percent of plasmatic mutations that existed in the original tumor.

The number of miscoding tumor mutations for patients PIT-063, PIT-069, PIT-075, and PIT-077 was 13, 18, 7, and 7, respectively. Of these, the fraction that LoFreq identified in plasma samples varied from zero to 100% (Figure 2, top panel). In particular, all tumor mutations were detected in the first plasma sample of PIT-075 and a large fraction in both samples of PIT-077, whereas for PIT-063 and PIT-069 few mutations were detected in the first plasma sample, and none in the second.

We also considered the problem from the opposite angle and asked how many of the variants called by the software did correspond to genuine tumor mutations. All software packages called an implausibly high number of variants (often several hundreds) and LoFreq was no exception. We attempted to identify somatic mutations among these by retaining only variants with a frequency lower than 30%, as variants that were more common than this were likely germline in nature. However, when we checked for the presence of these variants in leukocyte DNA, we found a large fraction of them, indicating that these were not genuine tumor mutations. We thus systematically sequenced leukocyte DNA in each patient and considered only low-frequency variants that were absent from leukocyte DNA as genuine somatic mutations. Among those identified in the first set of plasma samples, only 33–67% corresponded to mutations that had been found in the tumor (Figure 2, bottom panel). In the second set of plasma samples, a much lower fraction (0–8%) of presumably somatic mutations actually matched mutations found in the original tumor.

### Mutation Burden and Mutation Frequency

**Figure 3:**
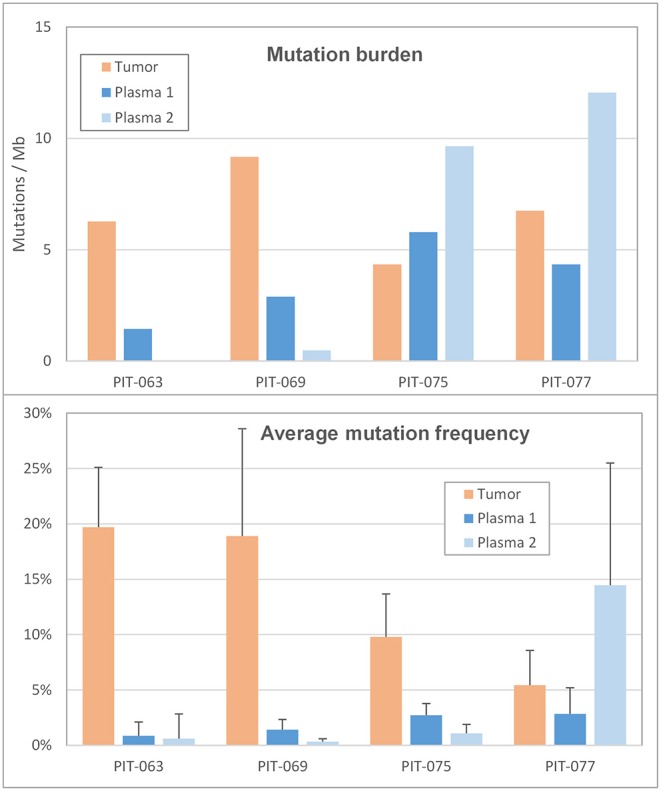
Mutation profile (**Top**) mutation burden in the tumor and in the two plasma samples, expressed as the number of miscoding mutations per megabase. (**Bottom**) Average frequency of the alternate allele in the tumor and in the two plasma samples. Error bars are the standard deviation.

We then calculated mutation burden by considering only miscoding mutations among those retained (i.e., called by LoFreq, with a frequency inferior to 30% and absent from leukocyte DNA), and normalizing this number for the size of the target region sequenced (Figure 3, top panel and Table 1). All 4 patients had TMB inferior to 10 mutations per megabase (Mbp); the corresponding mutation burden in the synchronous plasma samples was generally lower, inferior to 5 mutations per Mbp. The notable exception was patient PIT-075 who displayed a PTMB similar to, even slightly higher than his TMB. At the time of the second blood draw, after 3 months therapy, PTMB was zero for patient PIT-063 and very low for PIT-069, whereas it had notably increased for PIT-075, and reached almost 12 mutations per Mbp for PIT-077.

We also considered the impact of mutation frequencies (i.e., the percentage of mutant reads at a given genomic position) on mutation count, as low-frequency mutations, being harder to distinguish from background, are more likely to be missed. Among our 4 patients, PIT-063 had the lowest average mutation frequency in plasma, and also the lowest plasma mutation burden (Figure 3, bottom panel). Average mutation frequencies were slightly higher for PIT-069 and PIT-075, although still inferior to 3%. By contrast, patient PIT-077 showed low mutation frequencies in the first plasma sample but markedly elevated frequencies in the second, with an average of 14.5%. This phenomenon may be partly responsible for the higher mutation burden we calculated for this sample, by making it easier for the software to distinguish somatic mutations from background errors.

## Discussion

### Correlations With Clinical History

The 4 patients included in this pilot study illustrate different clinical and pathophysiological scenarios, in their initial situation and subsequent evolution.

Patient PIT-063 had a high TMB, yet the lowest initial PTMB. Most mutations identified in the tumor were present in plasma, but at very low frequencies (<1%), making it almost impossible for the software to distinguish them from background error noise. A compounding factor may be that this patient presented higher than average ccfDNA levels. Given the low mutation frequencies observed, it is likely that most of his circulating DNA did not originate from the tumor, and may just reflect a physiological fluctuation which, unfortunately, contributed to further dilute ctDNA and made mutation detection even more challenging. After 3 months of checkpoint inhibitor therapy, PTMB was down to zero. The clinical evolution was favorable, with a very good partial response at 3 months, maintained at 6 months.

This patient is a perfect illustration of the difficulties inherent to circulating DNA analysis: when a tumor does not release ctDNA into the blood stream, or does so in minute quantities, it is virtually impossible to detect tumor-born mutations, no matter how sophisticated the sequencing technique and how performant the analysis software. From a diagnostic/prognostic point of view, it is important to identify such situations, to distinguish them from real low-TMB cases. In other words, one needs a way to quantify the fraction of cell-free DNA that originates from the tumor. A possible method is to analyze the plasma for driver mutations identified in the tumor, e.g., epidermal growth factor receptor (*EGFR*) exon 21 p.L858R or Kirsten rat sarcoma (*KRAS*) exon 2 p.G12D. Such driver mutations are presumably an early event in tumorigenesis, thus they should be present in all tumor cells, and their frequency in plasma should thus fairly reflect the proportion of ctDNA. In cases when such a mutation was not identified in the tumor, or when no tumor tissue is available, another possibility would be to study cell-free DNA methylation. It has been shown that methylation markers at precise genomic locations can be used to determine the tissue of origin of circulating DNA (28) and in particular to identify ctDNA, as methylation is globally perturbed in cancer cells (29). This approach, however, has not yet been shown to be quantitative and could not be applied to our samples. We thus chose the former approach for PIT-063, using a *TP53* stop-gain mutation that was present at 33% in the tumor, but only at 0.9% in plasma, thereby confirming our hypothesis that very little ctDNA is present in this patient's ccfDNA.

Patient PIT-069 was similar to PIT-063, with low mutation frequencies and a PTMB largely inferior to his TMB. His PTMB had significantly decreased after 3 months of immunotherapy, as well as the average mutation frequency (the amount of ccfDNA was similar at both time points, ruling out a dilution effect). Yet, none of the mutations identified in this second plasma sample were found in the original tumor. This likely represents an example of clonal evolution within the tumor, with novel passenger mutations appearing in numerous subclones, and being detected at low frequencies in the plasma. The clinical evolution entailed a dissociated response, with a notable regression of some metastases at 3 and 6 months, yet the progression of others at 6 months. Treatment was continued, and ultimately, radiotherapy was used on the progressive lesions. The newly identified clones in the plasma may thus correspond to those involved in the progression of the refractory metastases.

Patient PIT-075 was the only one with an initial PTMB matching its TMB. Mutation frequencies in the plasma were the highest of the 4 patients, with average levels of ccfDNA, likely indicating that the tumor was releasing significant amounts of ctDNA in the blood flow. It should be noted that PIT-075 was the only patient enrolled to have squamous histotype. As such, the question arises as to whether the high ctDNA release and PTMB could be related to histology. While current data suggests similar PTMB between ADC and squamous histotypes, we did not find information comparing their ctDNA release (30).

After 3 months of therapy, PTMB had significantly increased, whereas the average mutation frequency had declined, thereby ruling out a detection bias. The clinical evolution mirrors the decrease in mutation frequency, with a very good partial response. There was no 6-month radiological evaluation, as it was declined by the patient, but he remains asymptomatic at 15 months after treatment initiation.

In this patient, mutation burden analysis in the plasma was successful and matched that in the tumor. Both values were largely inferior to 10 mutations/Mb, the cut-off used in the Checkmate 227 trial (12) as a threshold for high tissue mutation burden, but the latter study counted all mutations in the coding sequence (including synonymous) whereas we only considered miscoding mutations. Counting synonymous mutations for this patient would result in higher values: 4.8 for TMB and 8.7 for PTMB, the latter approaching the 10 mutations/Mb threshold. In patients treated with combination immunotherapy (anti PD-1 and anti-CTLA4 antibodies), with tumors expressing PD-L1 on <1% of cells, high TMB was associated with a 1 year progression-free survival rate of 45%, as opposed to 18% with low TMB. It is important to note that ultimately, this trial did not show any survival difference by TMB (11). A preplanned exploratory analysis from the MYSTIC trial showed an OS benefit in patients with PTMB > 16 mutations/Mbp in front-line combined anti-PD-L1 and anti-CTLA4 immunotherapy for advanced NSCLC (31). However, the Neptune trial, which prospectively assessed the role of PTMB in this same setting with the same treatment failed to show any predictive relevance of PTMB in the primary analysis (32). There appears to be a correlation between mutation burden and response in our patient. While this may be a coincidence, the decrease in mutational frequency suggests a dynamic role of PTMB.

Patient PIT-077 had the lowest amount of ccfDNA and one of the highest average mutation frequencies among our 4 patients. It can be assumed that the tumor was releasing a fair amount of ctDNA at the time of the first blood sampling. Her initial PTMB was 4.3 mutations/Mbp, but after 3 months of immunotherapy, the molecular situation had significantly worsened, with a high PTMB (12.1 miscoding mutations/Mbp), high mutations frequencies (14.5% in average) and very high levels of ccfDNA (73 ng/ml plasma). This likely indicates disease progression, with a tumor that accumulated novel mutations (only 8% were present in the initial tumor), and released large amounts of ctDNA into the blood flow. The clinical evolution supports the biological hypothesis, with clear disease progression at 3 months and cancer-related mortality at 6 months.

This patient was illustrative of a situation in which liquid biopsy proved superior to a traditional tumor biopsy, in that it allowed us to non-invasively detect a drastic increase in PTMB and in average mutation frequency after 3 months, which were in line with the poor, eventually fatal evolution of the patient.

### Lessons Learned

Although limited in the number of patients, this pilot study allowed us to draw important methodological conclusions pertaining to the analysis of mutation burden in the plasma.

First and foremost, the key to a valid analysis is reliable identification of genuine somatic mutations, originating from the tumor. This is a particularly difficult problem in the case of mutation burden analysis since, by definition, passenger mutations originate from distinct cells and are expected to be present only in small subclones within a tumor. As a result, one can expect mutation frequencies to be quite low in plasma, making it difficult to distinguish genuine mutations from PCR or sequencing errors.

There are several ways to address this issue. One is to reduce background noise via technical improvements to DNA sequencing or library synthesis. In this respect, the advent of molecular barcodes was a major step forward, as it allows the identification of sequencing mistakes and PCR errors occurring after the second PCR cycle. Yet, we found it insufficient in the case of mutation burden analysis. A second key factor is bioinformatic processing, and the development of highly efficient algorithms to discriminate mutations from background noise. Here also, despite the release of a number of specific software packages in the recent years, we did not find one that was fully efficient for the purpose of mutation burden analysis.

A critical point, in our experience, is the need to sequence leukocyte DNA. We observed that a simple mutation frequency filter is not sufficient to reliably identify somatic mutations. For one, because it is conceivable that a somatic mutation may reach a frequency approaching 50%, e.g., in advanced cases when a tumor is releasing large amounts of ctDNA. But more importantly, the bulk of cell-free DNA was shown to originate from hematopoietic cells (33) and it is known that even normal individuals can accumulate clonal mutations in various leucocyte lineages upon aging, a phenomenon known as clonal hematopoiesis (34). Although somatic, these mutations do not originate from the tumor and should not be tallied when calculating PTMB. The only way to exclude them from analysis it to perform two rounds of sequencing, one on plasma and one on leucocytes, obtained either from whole blood or from the buffy coat resulting from plasma preparation.

A second conclusion of this study is that mutation burden measurements in plasma rarely match those in the tumor. There could be several reasons for this: it may happen that a tumor releases very low amounts of ctDNA, as in the case of PIT-063, making it virtually impossible to identify tumor mutations in plasma. Furthermore, as the amount of ccfDNA originating from normal cells fluctuates rapidly, it may dilute ctDNA in various proportions depending when phlebotomy is performed. This is an important consideration to keep in mind if one intends to make use of PTMB for patient follow up over an extended period of time. A possible solution would be to perform several closely spaced blood draws (e.g., 3 or 4 in a day) and sequence them in parallel, or to select the one with the lowest ccfDNA yield for sequencing. This may, however, impose an extra burden on the patient and significantly increases the cost of the analysis.

Conversely, as many tumors (e.g., lung and kidney) are genetically heterogeneous, a surgical biopsy may not account for the whole collection of mutations a tumor comprises. It was shown that ctDNA analysis provides a more complete reflection of the mutational landscape than a surgical biopsy, both in the main tumor (35) and in eventual metastases (36). Thus, provided the above limitations are addressed, PTMB may prove a more reliable prognostic indicator than TMB.

Ultimately, whether mutation burden is predictive or not of response to ICPI treatments, the analytical complexity involved with the biomarker could limit its reproducibility and reliability. While mutation burden has potential, it currently does not deserve a role in therapeutic decision-making. Nonetheless, the potential value of PTMB during therapy, especially given the difficulty in interpreting radiological response to immunotherapy (37), remains to be better elucidated.

## Data Availability Statement

The datasets generated for this study are available on request to the corresponding author.

## Ethics Statement

The studies involving human participants were reviewed and approved by Local Ethical Committee in Geneva. The patients/participants provided their written informed consent to participate in this study.

## Author Contributions

AA, AF, TM, and TN designed the study. AA and AF provided the samples. LH performed sequencing. YC and TN performed bioinformatic analyses. AF and TN wrote the paper. All authors reviewed and approved the paper.

### Conflict of Interest

AA has received research funding from Boehringer Ingelheim, and has received compensation from Bristol-Myers Squibb, AstraZeneca, Merck Sharpe & Dohme, Takeda, Pfizer, Roche and Boehringer Ingelheim for participating on advisory boards. AF has received compensation from Roche, Pfizer, Astellas and Bristol-Myers Squibb for service as a consultant. The remaining authors declare that the research was conducted in the absence of any commercial or financial relationships that could be construed as a potential conflict of interest.
